# Thiamine: An indispensable regulator of paediatric neuro-cardiovascular health and diseases

**DOI:** 10.1007/s00431-024-05756-4

**Published:** 2024-09-13

**Authors:** Ysphaneendramallimoggala, Monalisa Biswas, Stanly Elstin Anburaj, Faiza Iqbal, Shrikiran A., Varashree Bolar Suryakanth, Leslie Edward S. Lewis

**Affiliations:** 1 Department of Paediatrics, Kasturba Medical College, Manipal, Manipal Academy of Higher Education, Manipal, Karnataka India 576104; 2 Department of Biochemistry, Kasturba Medical College, Manipal Academy of Higher EducationKasturba Medical College, Manipal, Manipal Academy of Higher Education, Manipal, Karnataka India 576104; 3 Department of Health Information, Prasanna School of Public Health, Manipal Academy of Higher Education, Manipal, Karnataka India 576104

**Keywords:** Thiamine, Thiamine pyrophosphate, Thiamine triphosphate, Neonates, Persistent pulmonary hypertension of newborn, Metabolic acidosis, Hypoxic ischaemic encephalopathy, Energy metabolism

## Abstract

The sustainable developmental goals emphasize good health, reduction in preventable neonatal and under-five mortalities, and attaining zero hunger. However, South Asian countries report a higher incidence of neonatal and under-five mortalities when compared to the Western world, many of which are attributed to maternal and perinatal micronutrient deficiencies. Isolated nutrient deficiency in the absence of calorie deficit poses a diagnostic challenge since such deficiencies present with acute multisystemic and enigmatic manifestations. Thiamine (vitamin B1) is a micronutrient of prime importance which exerts indispensable roles in energy metabolism. Deficiency of thiamine can lead to catastrophic consequences. This review provides insight into the biochemical actions of thiamine in energy metabolism, the compromised aerobic metabolism resulting from thiamine deficiency, and the crucial role of thiamine in the proper functioning of the nervous, cardiovascular, and immune systems. The review also explores the acute life-threatening consequences of thiamine deficiencies in neonates and infants and the speculative role of thiamine in other pathologies like encephalopathy, sepsis, and autism spectrum disorders. However, routine assessment of thiamine in pregnant women and neonates is yet to be implemented, due to the lack of affordable and automated diagnostic techniques, and the cost-intensive nature of mass spectrometry-based quantification. *Conclusion*: Physicians are recommended to have a low threshold for suspecting thiamine deficiency especially in vulnerable populations. Laboratory diagnosis of thiamine deficiency needs to be implemented as a standard of care, especially in endemic regions. Further, public health policies on food fortification, mandatory supplementation, and surveillance are imperative to eliminate thiamine deficiency-induced health hazards.

**Table:** 

What is Known: • *South Asian countries report a higher incidence of neonatal and under-five mortalities, many of which are attributed to maternal and perinatal micronutrient deficiencies.* • *Preventable causes of neonatal/ infantile deaths include birth factors (low birth weight, birth asphyxia), infectious diseases (pneumonia, diarrhoea, tetanus, tuberculosis, measles, diphtheria, malaria, acute infections), deficiency diseases and genetic diseases (vitamin & mineral deficiencies, IEMs, congenital heart disease, unexplained PPHN, SIDS etc).*
What is New: • *Acute thiamine deficiency presenting as multisystemic syndromes, has unfortunately been a long standing unresolved public health concern. However, accessible surveillance and diagnostic strategies remain elusive in most clinical settings.* • *Despite decades of reports and emerging guidelines, diagnosis of thiamine deficiency is often missed and policy mandates at national level are yet to be implemented even in endemic countries.* • *This review provides a comprehensive summary of the biochemical role of thiamine, its key functions and effects on major organ systems, the diagnostic gap, the enigmatic presentation of acute thiamine deficiency, the plausible role of thiamine in other pathologies and the preventive measures at individual and community level.*

**Supplementary Information:**

The online version contains supplementary material available at 10.1007/s00431-024-05756-4.

## Introduction

Nutrition plays a crucial role in shaping the “state of well-being” and exerts long-term effects on health and disease trajectories [1]. Micronutrients include vitamins, minerals, and trace elements which cannot be synthesized by the body; deficiencies are bound to interfere with normal physiological signalling [1]. Micronutrient deficiencies (in the absence of accompanying calorie deficit), also referred to as “hidden hunger”, are estimated to be afflicting approximately two billion people, globally, and have assumed an epidemic form in India [2–4]. The first 1000 days of an individual’s life play a pivotal role in determining the overall well-being of the child [5]. Maternal or infant micronutrient deficiencies during this phase can have serious consequences [5]. Nutritional deficiencies account for considerable mortalities specially in low-middle income countries (LMICs) [6].

Thiamine (vitamin B1), an underexplored water-soluble vitamin, plays an indispensable role in energy metabolism, neural functioning, and growth and development [7]. Dietary thiamine is absorbed in the small intestine by active transport and is stored in the liver in minimal quantities [7]. However, thiamine is characterized by a very short half-life which mandates a continuous dietary supply [7]. Though acute thiamine deficiency is life-threatening and is a long-standing forgotten/unresolved public health concern in LMICs, diagnostic strategies remain elusive. Further, the assessment of vitamin B1 as a standard of care is yet to be implemented even in endemic countries. Existing literature emphasizes on specific aspects of thiamine deficiency, majorly the overt deficiency manifestations, but a comprehensive review summarizing the biochemical and physiological roles and their direct impact on major organ systems, and the plethora of varying deficiency manifestations worldwide, is absent. This review aims to summarize the physiological and biochemical role of thiamine, the indispensable role of thiamine in the optimum functioning of the nervous and cardiovascular system, the systemic effects precipitated by thiamine deficiency, and strategies to aid the prevention of thiamine deficiency at the community level.

## Why are neonates and infants more vulnerable to thiamine deficiency?

The global prevalence of thiamine deficiency remains unavailable and hugely under-reported due to its enigmatic presentation (mimicking acute systemic illness), inaccessible laboratory assessment techniques, or the deficiency being masked under the blanket of malnutrition [8]. Thiamine deficiency is rare among healthy adults belonging to a middle or high socioeconomic status with cultural practices encouraging food diversity [9].

**Fig. 1:**
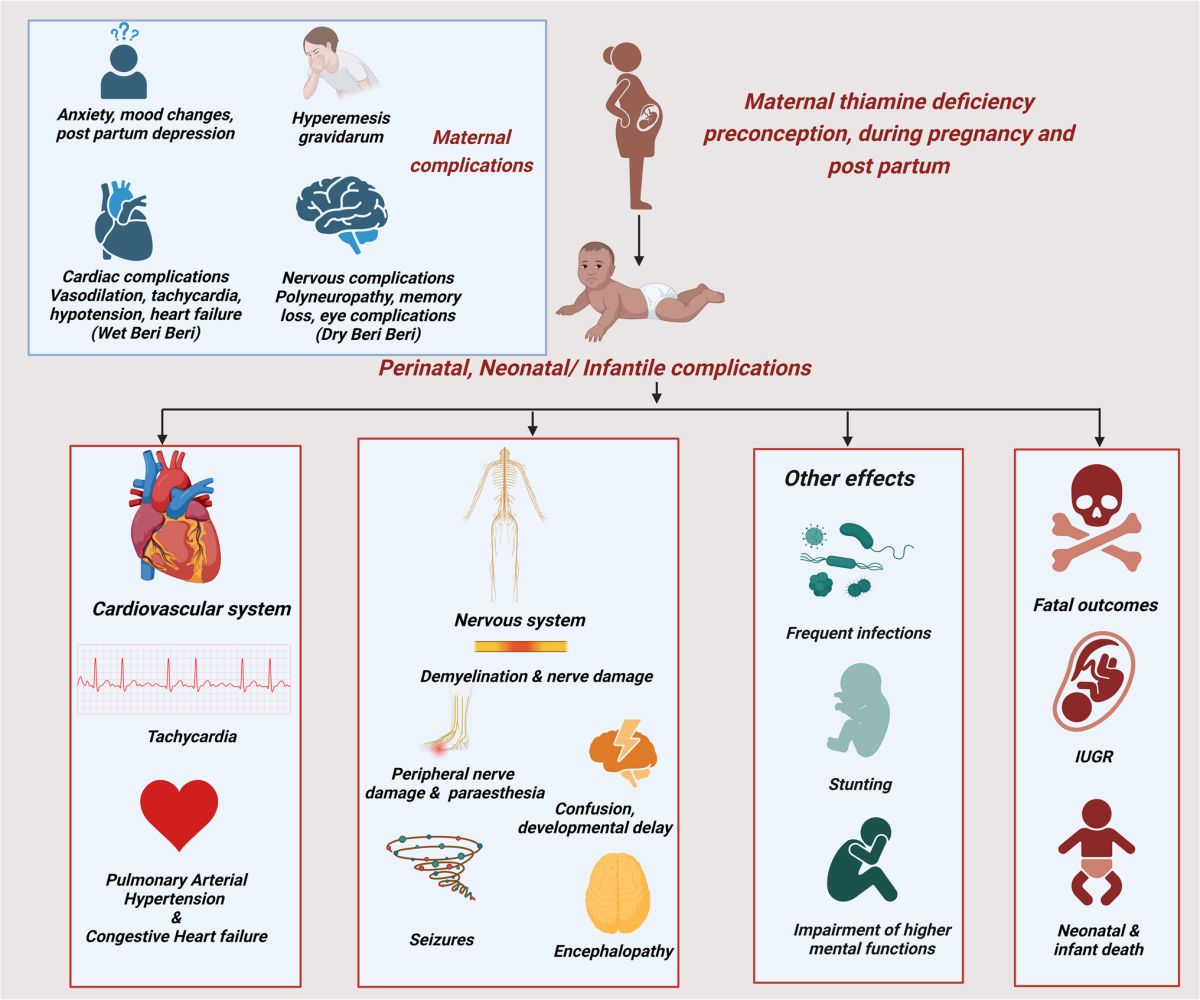
Neonatal, infantile, and maternal consequences of thiamine deficiency during pregnancy and early post-partum phases. This figure describes the multifaceted consequences of maternal thiamine deficiency on neonatal organ systems; the effects range from fatal to chronic and have a lasting impact on the infant. Further, deficiency of thiamine is also implicated in maternal complications during pregnancy [1, 10, 11, 67]. Original image. Created in Biorender

However, neonates and infants are highly susceptible to thiamine deficiency that is associated with a high fatality rate [8, 9]. The risk enhances multiple folds when neonates who are exclusively breastfed are born to mothers consuming refined cereals (polished rice, wheat) as their staple food, women practicing culturally specified intrapartum food avoidances and women experiencing persistent hyperemesis, malabsorption, or some form of systemic illnesses during pregnancy [1, 10, 11]. Since thiamine has a short half-life, the effect of maternal and breast milk thiamine deficiency is unmasked in early infancy, once the newborn becomes completely dependent on breast milk thiamine [8–11]. Figure 1 summarizes the neonatal and maternal consequences of thiamine deficiency during pregnancy and early post-partum phases.

## Reported incidence of neonatal and infantile thiamine deficiency

Robust data on the worldwide incidence of thiamine deficiency remains unavailable to date. Thiamine deficiency is reported to be endemic and widespread in low and middle-income countries and countries having rice as their staple diet which include South Asian countries (Cambodia, Laos, Nepal, India, and Myanmar) and West Africa [12–14]. High-income countries show an increased incidence of thiamine deficiency among children and adolescents with chronic illness being the primary reported reason of deficiency [14]. The tragic outbreak of infantile thiamine deficiency in Israel, in the year 2003, due to the introduction of a thiamine-deficient infant soy-based formula provides a glaring example of the global vulnerability to precipitation of acute thiamine deficiency [15]. India has a presumably high incidence of infantile thiamine deficiency [9, 16]. Studies have reported that a significant percentage of early infants presenting with respiratory distress and tachycardia have underlying thiamine deficiency [8]. The reported worldwide prevalence of thiamine deficiency is highly variable ranging from 13.4% in hospitalized children in Laos and 30% in Laotian children to 40% in malnourished children of Jamaica and Ghana [17]. Awareness of thiamine deficiency, its variable clinical spectrum, a low threshold for suspicion, and early thiamine supplementation can significantly improve the management of acutely presenting critically ill neonates, infants, and children [8, 16].

## Biochemical functions of thiamine

Thiamine is comprised of a “pyrimidine ring (2,5-dimethyl-6-aminopyrimidine) and a thiazolium ring (4-methyl-5-hydroxy ethyl thiazole) joined by a methylene bridge” [18]. The pyrophosphate ester form of thiamine, that is thiamine pyrophosphate (TPP) or thiamine diphosphate (TDP), is the active form of thiamine. TPP accounts for approximately 80% of the thiamine present in the circulation [18].

### Actions of thiamine pyrophosphate (TPP)

**Fig. 2:**
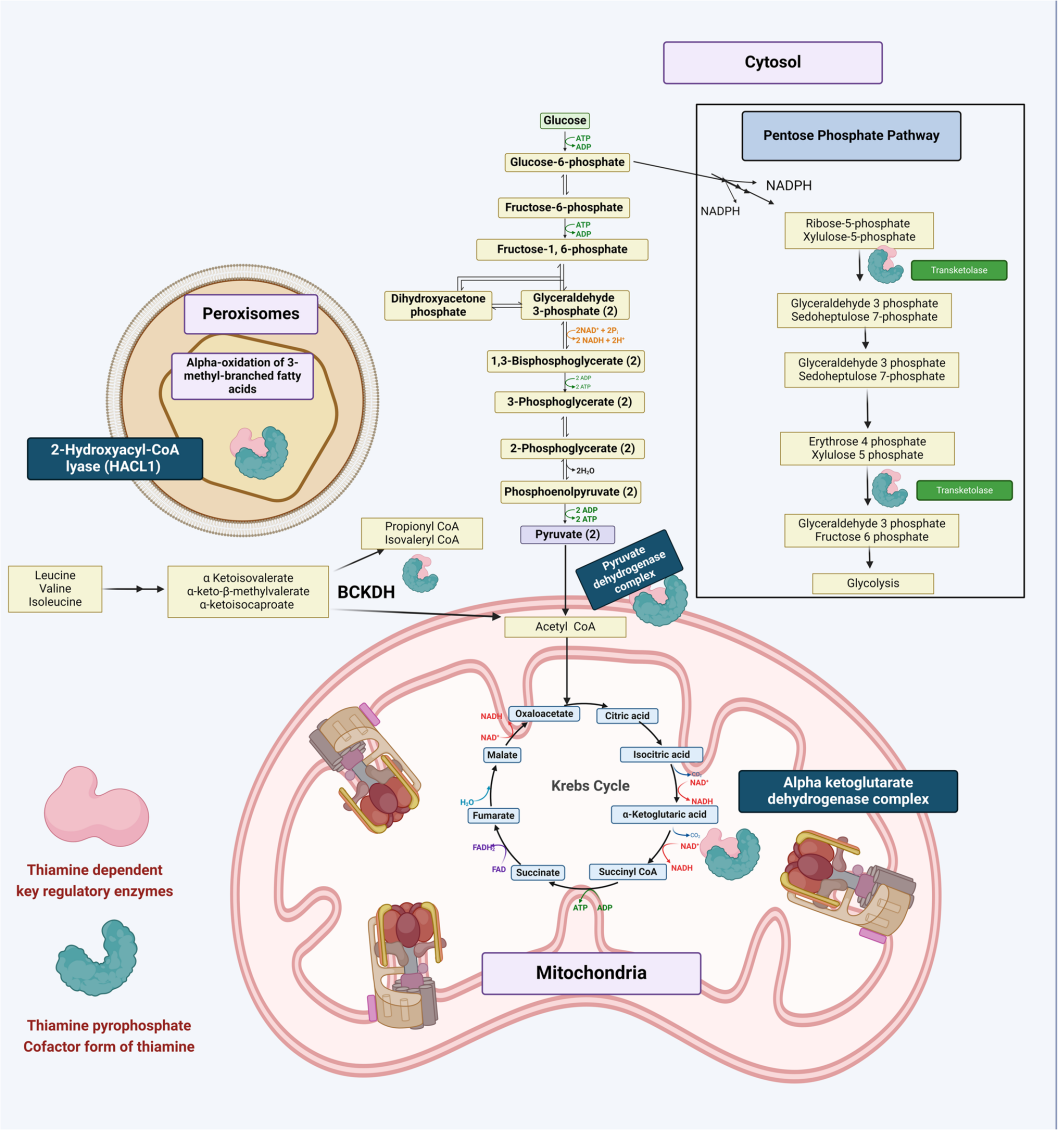
Central role of thiamine pyrophosphate in energy metabolism. This figure provides a schematic description of all thiamine-dependent key enzymes regulating energy metabolism thus providing an insight into the mechanism of compromised ATP production and lactic acidosis in thiamine deficiency [19–30]. Original image. Created in Biorender

TPP is intricately involved in the metabolism of carbohydrates, fats, and mitochondrial energetics. Figure 2 summarizes the key regulatory roles of TPP in energy metabolism.

Biochemical roles of TPP are as follows:

A) Pyruvate dehydrogenase complex: Pyruvate dehydrogenase complex is a multienzyme complex comprising three enzymatic domains (pyruvate dehydrogenase, dihydrolipoamide acetyltransferase, and dihydrolipoamide dehydrogenase) and four cofactors (TPP, coenzyme a, lipoamide, FAD, NAD) which catalyzes the conversion of pyruvate to acetyl CoA and is an indispensable enzyme in the metabolism of carbohydrates [19].

Deficiency of TPP leads to non-functioning of pyruvate dehydrogenase which interferes with the conversion of pyruvate to acetyl CoA and the entry of available carbohydrates to the Krebs Cycle [19, 20]. Accumulated pyruvate is diverted to the alternate anaerobic pathway of lactic acid production culminating in lactic acidosis [20]

B) Alpha-ketoglutarate dehydrogenase complex: Alpha-ketoglutarate dehydrogenase complex is a multienzyme complex of Krebs’s cycle [21]. It catalyzes the rate-limiting conversion of α ketoglutarate to succinyl CoA producing energy equivalents in the form NADH [21]. It comprises alpha-ketoglutarate dehydrogenase (E1), dihydrolipoamide S-succinyltransferase (E2), and dihydrolipoamide dehydrogenase (E3) [21].

Thiamine deficiency substantially compromises alpha-ketoglutarate dehydrogenase complex activity [22, 23]. An experimental study in the rodent model reported that thiamine deficiency resulted in a 52% reduction of the enzyme activity in the sub-medial thalamic nucleus [23].

C) Branched-chain α-keto dehydrogenase complex: The three branched-chain amino acids (BCAAs) are classified as essential amino acids with anabolic functions. This complex presents in the inner membrane of the mitochondria catalyzes the irreversible conversion of branched-chain keto acids to their corresponding acyl CoA esters [24]. It is a multienzyme complex comprising a heterodimeric decarboxylase component, a transacylase component, and a homodimeric enzyme 3 component and requires TPP, coenzyme A, lipoamide, FAD, and NAD as cofactors [24]. A study on the rat model reported that thiamine deficiency resulted in a significant reduction in the activity of this enzyme complex in the medial thalamus [25, 26]. The resultant exponential BCAA accumulation is reported to induce neuronal dysfunction and cell death [25, 26].
D) Transketolase: Transketolase is a major enzyme of the pentose phosphate pathway (PPP) or the hexose monophosphate shunt pathway (HMP) which is the major source of NADPH and pentose sugars [27]. Thiamine deficiency compromises transketolase activity [28, 29]. Studies on mice models have reported that thiamine deficiency-induced decrease in transketolase activity leads to impaired neurogenesis in the hippocampus affecting cognitive abilities [28, 29].
E) 2 Hydroxyacyl CoA lyase (HACL1): HACL is a TPP-dependent peroxisomal enzyme that aids in the oxidation of fatty acids like phytanic acid (3 methyl branched-chain fatty acids) and shortening of long-chain fatty acids [30]. Deficiency of thiamine blocks the activity of this enzyme compromising peroxisomal fatty acid metabolism [30].

### Action of thiamine triphosphate (TTP)

TTP constitutes a minor proportion of the thiamine reserves of the neuronal cells [31–33]. Supplementary file Table 1 summarizes the CNS functions of TTP [31–33, 68].

## Action of thiamine on organ systems

A) Central nervous system

**Fig. 3:**
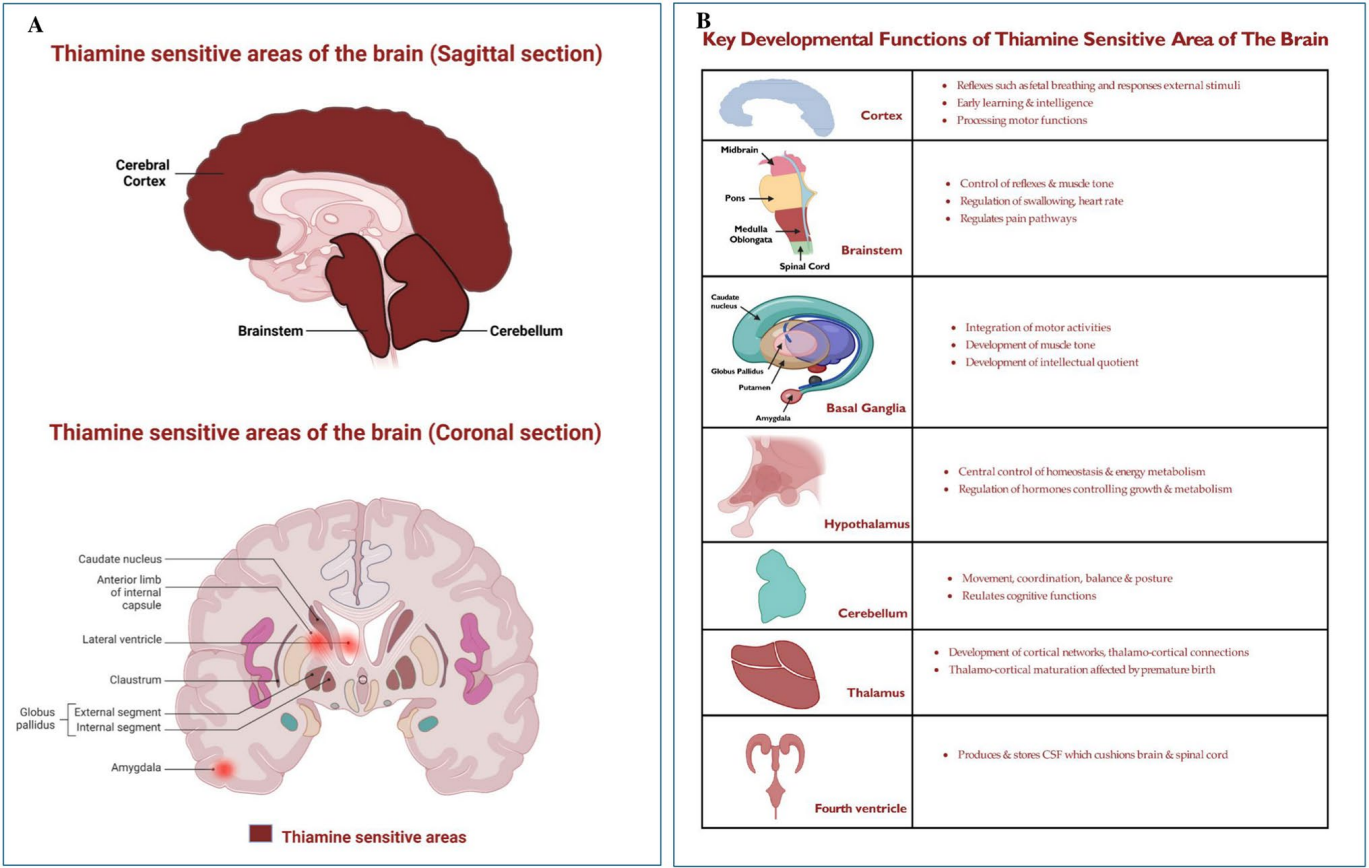
Role of thiamine in the central nervous system. **A** The thiamine-sensitive areas of the brain in the coronal (cerebral cortex, brainstem, cerebellum) and sagittal (Caudate nucleus, amygdala, fourth ventricle, globus pallidus, claustrum, internal capsule) sections and highlights the specific areas of the brain which will show mild to high sensitivity to thiamine deficiency [34–37]. **B** The thiamine-sensitive areas of the brain and their key role in the development and maintenance of the CNS functions and achievement of age-appropriate developmental milestones [34–37]. Original image. Created in Biorender

The nervous system primarily utilizes glucose for its energy requirement and hence is highly sensitive to alterations in the activity of energy metabolic pathways [9]. Deficiency of thiamine retards mitochondrial energy production limiting the activity of ion channels including the crucial Na^+^-K^+^ ATPase pump thereby affecting nerve impulses [9]. Thiamine deficiency also increases the oxidative damage and hinders the biosynthesis of fatty acid and nucleic acids crucial for myelination and cell division respectively [9]. The brain can be classified into thiamine-sensitive and thiamine-resistant areas; sensitive areas are reported to show considerable dependence [34]. Thiamine-sensitive areas in children include mammillary bodies, basal ganglia, and frontal lobes (Figure 3A) [35]. Figure 3B summarizes the key developmental roles of the thiamine-sensitive areas of the brain.

Thiamine deficiency leads to the loss of neuronal cells (Purkinje cells) [34]. Astrocytes are most vulnerable to thiamine deficiency showing altered astrocyte-endothelial interaction, release of inflammatory mediators, and downregulation of tPA and thrombomodulin in the brain endothelial capillary cells [34]. Further, astrocytes are the main site of lactate production. Persistent lactate generation leads to acidosis, stimulates the activity of the Na^+^–H^+^ exchanger, and increases the accumulation of Na ions exerting osmotic effects culminating in cellular oedema [34]. Increased intracellular sodium activates the Na^+^–Ca^2+^ exchanger; increased entry of calcium ions in the mitochondria of the astrocytes leads to cell death [34]. Thiamine deficiency also increases extracellular glutamate concentration and affects neurotransmission [34–37]. Thiamine deficiency is reported to increase the permeability of the brain via eNOS-induced oxidative stress and is implicated in haemorrhage and oedema [30]. Megadose supplementation of thiamine is also reported to inhibit thermal hyperalgesia [37, 38].

B) Cardiovascular system

The heart requires substantial amounts of energy equivalents to power its contractile and transport functions [39]. However, it has a limited capacity to store energy and hence requires high energy flux and depends largely on its cellular mitochondria [39]. Mitochondria aids in ATP generation, calcium homeostasis, and lipid synthesis; hence, mitochondrial dysfunction is strongly implicated in cardiomyopathy [39].

**Fig. 4:**
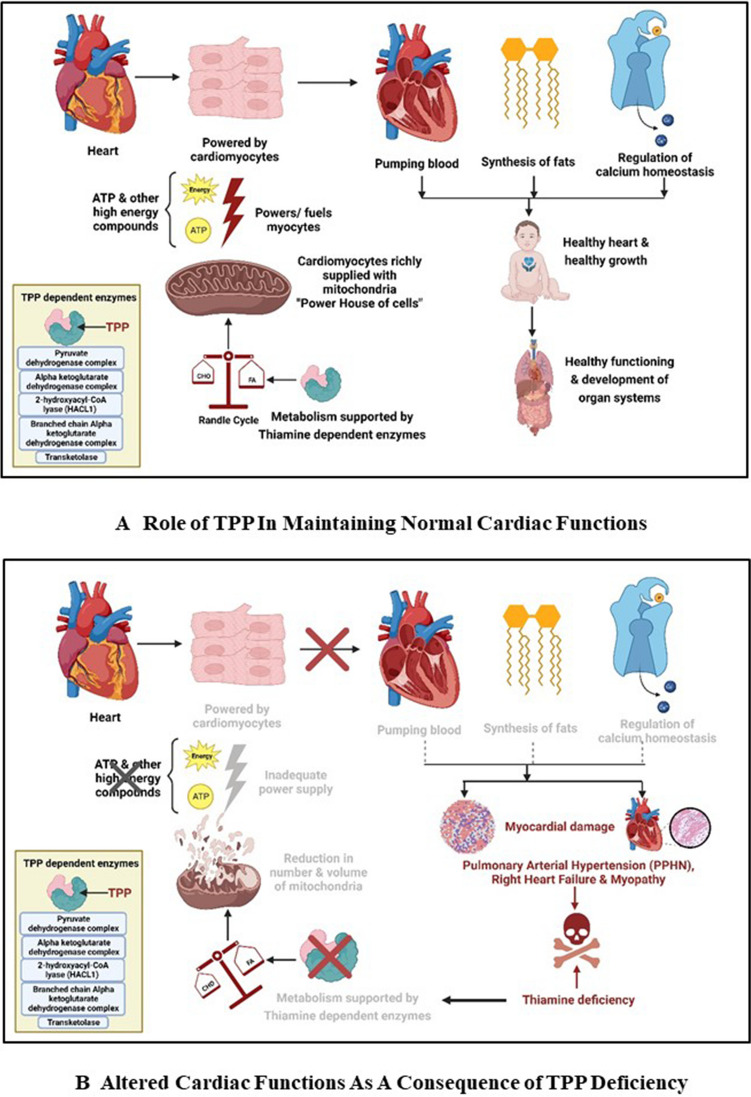
Role of thiamine in the regulation of cardiac function. **A** The key role of thiamine pyrophosphate in maintaining normal cardiac functioning which includes adequate energy supply to cardiomyocytes ensuring proper pumping function of the heart, synthesis of fats, regulation of calcium homeostasis, etc. Deficiency of thiamine pyrophosphate and the resultant faulty energy metabolism results in improper cardiac functions leading to right ventricular hypertrophy, pulmonary arterial hypertension, and heart failure and may lead to fatal consequences if left untreated. **B** These catastrophic consequences of untreated thiamine deficiency on cardiac functioning [41–43]. Original image. Created in Biorende

Thiamine deficiency-induced inhibition of the key regulators leads to impairment of ATP production and compromises mitochondrial functions [40]. Thiamine deficiency has been implicated in hypertrophy of the heart, heart failure with preserved ejection fraction (HFpEF), and lactic acidosis (Cardiac and Shoshin beriberi) (Figure 4) [39–41].

C) Immune system

Current studies are exploring the role of thiamine in the regulation of the immune system and the hypothesis that thiamine deficiency causes hyperreactivity of the immune system by activation of its attack arm [42, 43]. The various effects of thiamine on the immune system can be summarized in Table 1.

**Table 1:** Role of thiamine in regulating immune functions

**Target action**	**Role of thiamine**
Hemin dependent oxygenases [42, 43, 69]	a) Regulation of intracellular adhesion molecule activity which mediates immune reactions
Infection prevention [42, 43, 69]	a) Elimination of infections through regulation of PPAR-γ b) Activation of macrophage maturation and its microbicidal activity via IL6, TNF alpha cascades
Cellular immunity [42, 43, 69]	Thiamine antagonist reported to: a) Prevent the activation of cytosolic phospholipase A2 and inhibit the regulatory enzymes of cyclo-oxygenase pathway b) Retarded apoptosis by downregulating the expression of pro-apoptotic Bcl2, activation of caspase 3, PARP cleavage, and the release of cytochrome c c) Prevent activation of NF-κB
Tumour suppression [42, 43, 69]	Inhibition of p53 activity
Gut microbiota [42, 43, 69]	a) Aids the survival of symbiotic beneficial gut microbiota b) Deficiency reported to alter the relative abundance of Ruminococcaceae c) Essential cofactor for the generation of butyrate, a SCFA generated by the gut microbiota
Antioxidant activity [42, 43, 69]	a) Prevents oxidation of sulfhydryl groups b) Protects neutrophils from oxidative damage

## Diagnosis of thiamine deficiency

The gold standard for the assessment of circulating thiamine pyrophosphate is liquid chromatography-tandem mass spectrometry [13]. Studies have also reported HPLC-based methods for the assessment of TPP and other forms of thiamine [13]. Thiamine status can also be assessed indirectly by measuring erythrocyte transketolase activity through UV spectroscopy [13]. A study by Wani et al. has proposed that cranial ultrasonography reveals hyper-echogenicity of basal ganglia diagnostic of thiamine deficiency-induced encephalopathy with high sensitivity and specificity [44]. However, accessibility to these tests remains a challenge, especially in resource-constrained settings.

## Consequences of thiamine deficiency

A) Metabolic acidosis

Thiamine deficiency can lead to compromised energy metabolism and lactic acidosis [43]. Often misdiagnosed as sepsis due to its variable presentation, the resultant delay can lead to neonatal death even before diagnosis [45]. Multiple case reports have presented metabolic acidosis and associated cardiac complications in neonates and infants on TPN [14]. These patients have shown dramatic improvements in response to megadose intravenous thiamine supplementation [14] (Table 2).

**Table 2:** Recent reports on infantile thiamine deficiency presenting as acute metabolic acidosis

Research group, year of publication	Study population	Presenting symptoms	Response to thiamine
Hasan et al., 2023. [45]	Premature male infant (born at 30 weeks of gestation) on TPN	Lactic acidosis	A single dose of 50 mg IV thiamine resulted in drastic improvements within 6 h
Ozdemir et al., 2018. [70]	Premature low birth weight infant on 2 weeks TPN	Uncompensated metabolic acidosis	Dramatic resolution within 6 h of IV thiamine
Salvatori et al., 2016. [71]	2 preterm infants on prolonged TPN	Lactic acidosis with cardiac manifestations	150 mg IV thiamine led to drastic resolution of acidosis
Qureshi et al., 2016. [72]	23 exclusively breast-fed infants from Kashmir valley	Acute Metabolic acidosis	Dramatic resolution of symptoms in response of megadose (100 mg IV thiamine)
Oguz et al., 2011. [73]	Preterm infant (35 week gestational age)	Metabolic acidosis within 24 h of initiating TPN	-
Thauvin et al., 2004. [74]	11 neonates on un-supplemented TPN	Tachycardia, haemodynamic distress, refractory metabolic acidosis	Regression of symptoms in 4 neonates within few hours of administration of IV/IM thiamine, death of 7 infants

Studies suggest that intravenous dextrose in acutely ill patients with underlying thiamine deficiency causes aggravation of symptoms due to further depletion of thiamine [46]. Glucose load increases cellular demand of thiamine secondary to aggravation of blockage in mitochondrial metabolism, further enhancing lactic acidosis [46]. Borderline thiamine deficiency thus aggravates into an acute life-threatening one [46]. As per guidelines, intravenous thiamine supplementation of a minimum of 3 days should be given to all ICU patients suspected to have deficiencies [47]. Further, it is suggested to employ the thiamine challenge test in patients with unexplained illness, especially in endemic countries [48, 49]. A megadose of 100 mg of IV thiamine for 3 days followed by a 10 mg/day maintenance dose for 7 days has been reported in multiple studies without any evidence of thiamine-induced toxicity [9, 45, 48–51].

B) Pulmonary arterial hypertension (PAH)

Neonatal and infantile PAH have been reported in approximately 10% of infants with respiratory failure [50]. Thiamine deficiency-associated infantile PAH has been rampantly reported from selected regions of India. Infants usually present with right heart failure and acute metabolic acidosis within 3 to 4 weeks after birth to a period of 9 months [48, 51]. Thiamine deficiency leads to energy failure (Fig. 2); the “tired and damaged” myocardium performs suboptimal pumping, which increases end-diastolic pressure causing right heart dilation and PAH [52]. Further, reactive oxygen species exert a powerful vasoconstriction effect altering vascular resistance thus aggravating PAH [52]. Numerous case reports from Kashmir Valley, Karnataka, and Telangana have reported case series of infants presenting with thiamine-responsive PAH, metabolic acidosis, and cardiogenic shock [51] (Table 3).

C) Encephalopathy

**Table 3:** Recent reports on infantile thiamine deficiency presenting as severe PAH

Research Group, year of publication	Study population	Presenting symptoms	Response to thiamine
Sastry et al., 2021. [49]	250 exclusively breastfed infants from Bangalore with mothers on customary post-partum restricted diets	Tachycardia, poor feeding, severe PAH	231 infants (92%) responded dramatically to IV thiamine of 100 mg/day
Panigrahy et al., 2020. [51]	4 exclusively breastfed infants (two to four months old), with polished white rice as a staple diet in mothers	RDS, severe PAH	Resolution of symptoms with IV thiamine (100 mg)
Bhat et al., 2017. [75]	29 exclusively breastfed infants from Srinagar	Severe PAH	Complete resolution with IV thiamine (100 mg/kg) for 3 days
Rao et al., 2010. [76]	55 exclusively breastfed infants born to mothers belonging to low SES with rice as a staple diet	PAH, cardiomegaly	Complete resolution with 75 mg of IM thiamine twice a day for 5 days

Thiamine deficiency can manifest as acute encephalopathy [16, 53]. Countries where polished rice is the staple diet report high incidences of neonates presenting with severe unexplained encephalopathy, metabolic acidosis, shock, and mortality [16, 53]. The CDC reported three cases of death due to refractory lactic acidosis in TPN patients (without vitamin supplementation) triggered by acute thiamine deficiency [54]. Autopsies from two of the patients showed lesions suggestive of acute thiamine deficiency [55]. Table 4 summarizes the recent reports of infantile thiamine deficiency presenting as an acute encephalopathy.

**Table 4:** Recent reports on infantile thiamine deficiency presenting as encephalopathy

Research Group, year of publication	Study population	Presenting symptoms	Response to thiamine
Kornreich L. et al., 2005. [35]	6 infants within 10 months exclusively fed on soy-based formula devoid of thiamine	Encephalopathy, GI symptoms, nystagmus, seizures	Improvements observed with IM thiamine
Qureshi U.A. et al., 2021. [56]	43 thiamine deficient infants presenting as distinct classifiable presentations	GI symptoms, bilateral ptosis, encephalopathy	Treatment modality not mentioned
Rakotoambinina B. et al., 2021. [14]	389 cases of paediatric thiamine deficiency reported from high-income countries	Encephalopathy, lactic acidosis	IV thiamine as per clinical judgement
Pradhan D. et al., 2021. [77]	153 infants less than 2 years presenting with acute encephalopathy	Encephalopathy, respiratory failure	IV thiamine as per clinical judgement, with significant improvement, death of 59 infants, none of whom received supplementation
Narasimha R.S. et al., 2008. [57]	166 exclusively breast-fed infants born to mothers on rice/ rice soup as a staple diet belonging to low socioeconomic status	Encephalopathy, impaired consciousness, seizures, severe respiratory deficits	Thiamine supplementation (200–300 mg/day)

Infantile thiamine deficiency usually presents in one of the three above-discussed forms, that is cardiac beriberi characterized by severe acidosis, pulmonary arterial hypertension, and infantile encephalitic beriberi/pseudo-meningitic beriberi (Wernicke’e encephalopathy) [56–58].

D) Long-term consequences of thiamine deficiency

Long-term follow-up studies have reported that neonates or infants surviving acute manifestations of thiamine deficiency exhibit lasting effects which include neurological and cardiovascular deficits [8, 59]. Neurological manifestations include abnormalities in gross, grapho-, and fine motor skills, ataxia, seizures or compromised neuromotor functions (including quadriplegia, and hemiparesis), compromised vision (ophthalmoplegia, nystagmus), swallowing disorders, aphonia, auditory neuropathy, cognitive deficits, and speech and learning disorders [8, 59]. Reported long-term cardiovascular effects of severe thiamine deficiency include cardiomyopathy and non-resolving progressive heart failure in the absence of aggressive interventions [8, 13].

## Promising role of thiamine in other etiologies

A) Sepsis

Sepsis remains a challenging spectrum and a leading cause of neonatal mortality [60]. Recent studies are exploring the supportive role of thiamine in sepsis outcomes and the efficacy of combined hydrocortisone, ascorbic acid, and thiamine (HAT) regimen [61]. Thiamine deficiency in sepsis can be triggered by the enhanced metabolic demands during critical illness, effects of drugs, and administration of IV dextrose [46, 62]. It is recommended (K-24 proposal, Beth Israel Deaconess Medical Center, Boston) to initiate megadose IV thiamine as an “adjunct metabolic resuscitator” in all patients presenting with septic shock [46, 62]. However, few studies have failed to observe significant clinical improvement in response to megadose thiamine supplementation; patients with baseline deficiency of thiamine show significant improvement, making the data inconclusive and warranting further research [63].

B) Pathologies documenting speculative role

The role of thiamine deficiency has been explored and implicated in a plethora of other pathologies (SIDS, HIE, ASD) where concluding evidence regarding the causal relationship is yet to be elucidated [9, 64–66, 78–82] (Supplementary File Table 2).

## Measures to prevent thiamine deficiency

According to WHO [58] and current recommendations from field experts [13], a few measures to prevent thiamine deficiency include the following:

- Food fortification: Public health initiatives to fortify thiamine in appropriate food vehicles. Suggestions on the feasibility of biofortification through the genetic engineering of plants have also been explored [13].
- Thiamine supplementation as a standard of care: The Infantile Beriberi project of Myanmar is a benchmark interventional initiative implemented by the policy makers, which specifies mandatory thiamine supplementation (10 mg/day) to pregnant and lactating women. It is recommended that countries endemic to thiamine deficiency adopt this model in addition to their existing mandatory iron-folic acid supplementation in pregnant women [13].
- Improved surveillance: Countries endemic to thiamine deficiency should implement strategies to ensure mandatory assessment of circulating thiamine in pregnant women, neonates, and early infants (similar to mandatory TSH evaluation in newborn) and in populations with high vulnerability. A case series from Manipal, Karnataka, describes acute neurological manifestations (CT characteristic of Wernicke’s encephalopathy) in four women attributed to hyperemesis gravidarum-induced acute thiamine deficiency; complete resolution of symptoms was achieved with 1000 mg/day IV thiamine supplementation for 3 days [13, 67].
- Addressing malnutrition and food diversification: Consumption of diverse foodstuffs, especially a diet rich in legumes and vegetables, prevents thiamine deficiency [13, 58]. Culturally prevalent rigid post-natal maternal dietary restrictions have been attributed to neonatal and infantile thiamine deficiency in South Asian countries [13, 58].
- Adoption of cooking practices curtailing thiamine loss:
  Using parboiled rice, reduced prewashing of rice (with cold water), and ensuring cooking rice in the optimum amount of water to avoid discarding excess water, washing whole vegetables prior to cutting [13, 58].
  Prolonged cooking durations or recurrent heating and storage of cooked food or prolonged storage of even raw foods should be avoided [13, 58].
- Deactivation of anti-thiamine factors:
  Adequate precooking of thiaminase-rich foods like fish and meats (heat denatures thiaminase) [58, 67].
  Avoiding consumption of tea, coffee, blueberries, and other polyphenol and tannin-rich foods immediately after meals to ensure adequate absorption, citrus fruits can be consumed immediately after meals to aid absorption [58, 67].

Tragically, deficiency of thiamine is a long-standing (earliest reports dating back to approximately six decades) yet a forgotten and often neglected entity in public health. This review attempts to comprehensively summarize the crucial metabolic/physiological roles of thiamine and the pathological effects of thiamine deficiency. The review also provides a summary of therapeutic strategies and the imperative need for having a low threshold for suspecting thiamine deficiency, especially in vulnerable groups. Initiating a thiamine challenge test in the absence of diagnostic facilities can be a game changer in the management of acute crisis. Further, it is important that priority public health policies are made/ revised to accommodate for preventive standard of care strategies which could aid in a significant reduction in the incidence of acute thiamine deficiency.

## Conclusion

Thiamine is a pivotal water-soluble vitamin exerting a significant effect on energy metabolism and is critical for foetal, neonatal, and early infant development. Deficiency of thiamine can have catastrophic consequences. However, the lack of easily accessible diagnostic assays for thiamine and the enigmatic presentation of thiamine deficiency associated with acute illness make it a diagnostic challenge. It has been recommended to have a low threshold for thiamine supplementation in endemic regions and initiate a thiamine challenge test. Abrogation of symptoms in response to thiamine challenge is diagnostic of acute life-threatening deficiency. Further, there is an imperative need to develop simple, reliable, and affordable techniques to assess circulating thiamine levels and establish geography and age-specific biological reference intervals for thiamine. Assessment of breast milk thiamine levels could also identify high-risk neonates and infants and aid in prophylactic supplementation. Public health policies mandating fortification and surveillance along with community awareness on healthy dietary practices could serve as benchmark preventive strategies. Appropriate and comprehensive measures from clinicians, basic scientists, and policy makers can bring about drastic improvements in thiamine deficiency-associated fatalities and morbidities and may successfully eradicate thiamine deficiency-associated hidden hunger.

## Supplementary Information

Below is the link to the electronic supplementary material.

Supplementary file1 (DOCX 14 KB)

Supplementary file2 (DOCX 17.7 KB)

## Data Availability

No datasets were generated or analysed during the current study.

## Abbreviations

ASD: Autism spectrum disorder
ATP: Adenosine triphosphate
BCAAs: Branched-chain amino acid
CDC: Centres for Disease Control and Prevention
CoA: Coenzyme A
eNOS: Endothelial nitric oxide synthase
FAD: Flavin adenosine dinucleotide
HMP: Hexose monophosphate pathways
HACL: 2 Hydroxyacyl CoA lyase
HIE: Hypoxic ischemic encephalopathy
HIF-1α: Hypoxia inducible factor 1 alpha
HFpEF: Heart failure with preserved ejection fraction
LMICs: Low-middle income countries
NAD: Nicotinamide adenine dinucleotide
NADH: Reduced Nicotinamide adenine dinucleotide
PAH: Pulmonary arterial hypertension
PGI-R: (Parent Global Impressions—Revised)
PPAR-γ: Peroxisome proliferator-activated receptor gamma
PPP: Pentose phosphate pathway
PPHN: Persistent pulmonary hypertension of newborn
RCT: Randomized clinical trial
SIDS: Sudden infant death syndrome
TDP: Thiamine diphosphate
TPN: Total parenteral nutrition
TPP: Thiamine pyrophosphate
