# Supplementary material for: Thiamine: An indispensable regulator of paediatric neuro-cardiovascular health and diseases
Source: Eur J Pediatr. 2024 Sep 13;183(11):4597–610. doi: 10.1007/s00431-024-05756-4 (PMC11473601; doi:10.1007/s00431-024-05756-4)
Supplement: Supplementary file 1 — Supplementary file1 (DOCX 14 KB) [file 431_2024_5756_MOESM1_ESM.docx]

**Table 1: Role of TTP in the regulating the functions of the central nervous system**

| **Function** | **Mechanism of TTP action** |
| --- | --- |
| Membrane conductance [31-33] | Release of acetylcholine at the nicotinic acetylcholine synaptic receptors |
| Activation of chloride channel [31-33] | a) Positive correlation reported between TTP concentration and chloride permeability in rat brain  b) Thiamine antagonist reported to inhibit chloride uptake |
| Acts as a phosphate donor [31-33, 69] | Acts as a phosphate donor for endogenous kinases in the brain  Example: TTP specific nicotinic acetylcholine receptors in the post synaptic membrane |
