# Supplementary material for: Thiamine: An indispensable regulator of paediatric neuro-cardiovascular health and diseases
Source: Eur J Pediatr. 2024 Sep 13;183(11):4597–610. doi: 10.1007/s00431-024-05756-4 (PMC11473601; doi:10.1007/s00431-024-05756-4)
Supplement: Supplementary file 2 — Supplementary file2 (DOCX 17.7 KB) [file 431_2024_5756_MOESM2_ESM.docx]

**Supplementary file Table 2: Other pathological conditions where thiamine deficiency may be implicated**

| **Pathological condition** | **Research Group, year of publication** | **Possible reported role of thiamine deficiency** | **Research Group, year of publication** | **Contradictory evidence/ hypothesis reported** |
| --- | --- | --- | --- | --- |
| **Sudden Infant Death Syndrome (SIDS)** | Jeffrey HE et al, 1985. [79]  Barennes H et al, 2015. [80] | A) High incidence thiamine deficiency in ‘near-miss' SIDS infants, their mothers, older siblings.  B) Luang Namtha province reports prevalence of thiamine deficiency with supplementation producing dramatic response in approximately 90% of infants presenting with acute cardiac failure (which may lead to undiagnosed death of infant in absence of supplementation). | Davis RE et al, 1982. [81]  Lonsdale D et al, 2015. [82] | A) Five-fold increase in serum thiamine in SIDS patients demand re-exploration of the hypothesis.  B) Risks of SIDS multifactorial C) Attributed to the additive effects of:  i. Genetic risk  ii. Maternal/ infantile stress  iii. Thiamine metabolism implicating transporters, toxins with thiaminase, thiamine pyrophosphokinase |
| **Neonatal Hypoxic Ischemic Encephalopathy (HIE)** | Allen KA et al, 2011 [65], Varghese B et al, 2016 [66]. | A) HIE induced brain injury produces similar lesions as thiamine deficiency & Wernicke’s encephalopathy.  B) High doses of parenteral thiamine might be beneficial in HIE | Sechi GP et al, 2021. [67] | Clinical trials report thiamine supplementation alone fails to achieve resolution and neuroprotection a substantial percentage of neonates with HIE |
| **Autism Spectrum Disorders (ASD)** | Dhir S et al, 2019. [9] | A) Lower thiamine levels reported in ASD  B) Subset of ASD children have shown compromised pyruvate dehydrogenase activity | Anwar A et al, 2016. [83]  Dhir S et al, 2019. [9] | A) Quantification of thiamine metabolites 27 consecutive ASD children reported that none of them had thiamine deficiency  B) Lack of evidence to propose that thiamine deficiency might be a significant/ sole contributor of ASD, however it could certainly exert an additive role |
